# Exploring the Diversity and Function of the SPFH‐Domain Containing Proteins in *Pseudomonas aeruginosa*

**DOI:** 10.1111/1758-2229.70340

**Published:** 2026-04-16

**Authors:** Víctor Manuel García‐Maldonado, Claudia Rodríguez‐Rangel, Dimitris Georgellis, Adrián F. Alvarez

**Affiliations:** ^1^ Departamento de Genética Molecular, Instituto de Fisiología Celular Universidad Nacional Autónoma de México México City México

## Abstract

The SPFH‐domain containing proteins are widely conserved membrane‐associated factors proposed to organise membrane microdomains and thereby regulate key cellular processes. In *Pseudomonas aeruginosa* PA14, we identified nine such proteins (HflK, HflC and PaFlo3–PaFlo9) that display remarkable sequence divergence, genomic variability and limited phylogenetic conservation. Functional analyses of single and multiple SPFH mutants showed that these proteins are not essential for growth, biofilm formation, swimming and swarming motility, oxidative stress resistance or virulence in the *Galleria mellonella* infection model, although distinct slight phenotypic effects were observed in specific genetic backgrounds. Phylogenetic comparisons showed that none of the PaFlo proteins cluster with canonical bacterial flotillins such as FloA or FloT from *Bacillus subtilis* or FloA from *Staphylococcus aureus* , ruling out specific orthology. Moreover, most PaFlo proteins appear to not have clear orthologs in other γ‐Proteobacteria, indicating that they may be narrowly distributed in these bacterial genomes. The absence of conserved genomic context and operon organization further supports functional diversification rather than redundancy. These findings indicate that SPFH proteins are largely dispensable for *P. aeruginosa* viability and virulence under laboratory conditions and likely fulfil context‐dependent or niche‐specific roles.

## Graphical abstract

We characterised nine SPFH‐domain containing proteins in *Pseudomonas aeruginosa* PA14 and found remarkable sequence divergence and scattered evolutionary distribution. Functional analyses showed that single or multiple deletions had no detectable impact on growth, motility, biofilm formation, stress resistance or virulence under laboratory conditions. These results indicate that PaFlo proteins are dispensable for core physiology and may fulfil specialised, niche‐specific roles in Gram‐negative bacteria.

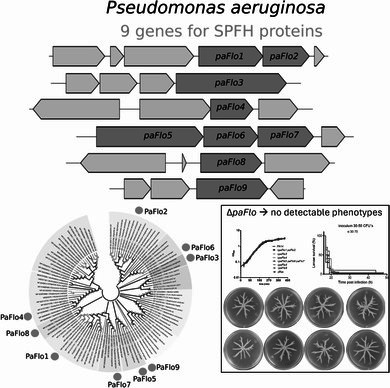

## 1 Introduction

SPFH‐domain containing proteins (named after Stomatin, Prohibitin, Flotillin and HflK/C) constitute a widely conserved family of membrane‐associated proteins found across all domains of life, including archaea, bacteria and eukaryotes (Rivera‐Milla et al. 2006; Hinderhofer et al. 2009). In eukaryotic cells, these proteins are integral components of lipid rafts, where they contribute to the recruitment and anchoring of membrane proteins to the cortical actin cytoskeleton (Langhorst et al. 2005, 2007; Browman et al. 2007). In bacteria, SPFH proteins are typically membrane‐associated and are proposed to function as scaffolding elements that organise proteins involved in signal transduction, stress responses and metabolic regulation (Lopez and Koch 2017; Alvarez and Georgellis 2019). In Gram‐positive species such as *Bacillus subtilis* and *Staphylococcus aureus* , flotillin‐like proteins FloA and FloT modulate membrane architecture, coordinate the assembly of multi‐protein complexes and contribute to virulence and adaptation to environmental stress (Schneider, Klein, et al. 2015; Dempwolff et al. 2016; Koch et al. 2017; Mielich‐Süss et al. 2017; Ukleja et al. 2024). In bacterial genomes, SPFH proteins are often encoded within operons together with NfeD encoding genes. NfeD proteins, initially identified in *Sinorhizobium meliloti* as a factor enhancing plant nodulation (García‐Rodríguez and Toro 2000), are exclusive to prokaryotes. While the specific roles of NfeD proteins remain unclear, it has been proposed that they may enhance SPFH protein function by promoting oligomerization (Tan et al. 2024; Ukleja et al. 2024). Notably, NfeD related proteins exist in short and long forms, the latter containing a ClpP protease domain. Yokoyama and Matsui (2005) reported that the SPFH protein PH1511 of *Pyrococcus horikoshii* undergoes C‐terminal cleavage catalysed by PH1510, a long NfeD homologue encoded within the same operon, and suggested a regulatory role in ion channel aperture (Yokoyama and Matsui 2005).

A common feature of SPFH‐domain proteins is their enrichment in detergent‐resistant membrane (DRM) fractions. In eukaryotes, this property is attributed to the presence of tightly packed lipids in liquid‐ordered phases, such as those found in lipid rafts, which resist solubilisation by non‐ionic detergents (Magee and Parmryd 2003). Proteomic analyses of DRM fractions from the plasma membranes of *B. subtilis* , *S. aureus* , *Borrelia burgdorferi* , *Helicobacter pylori* and *Escherichia coli* have similarly revealed enrichment of SPFH proteins in DRM fractions (López and Kolter 2010; Yepes et al. 2012; García‐Fernández et al. 2017; Hutton et al. 2017; Toledo et al. 2018; Guzmán‐Flores et al. 2019). However, it remains unclear whether this enrichment reflects specific lipid associations or the formation of high‐order membrane‐bound oligomeric assemblies. In *E. coli* , the best‐characterised SPFH proteins are HflK and HflC, which assemble into a membrane‐associated complex with the essential protease FtsH. This complex modulates FtsH proteolytic activity, contributing to membrane protein quality control and turnover (Kihara et al. 1996, 1997). More recently, the HflKC complex was shown to bind to and recruit the sensor kinase BarA to the cell poles, resulting in its inactivation and subsequent downregulation of BarA‐dependent gene expression during the stationary phase of growth (Contreras et al. 2023). Importantly, HflKC‐mediated regulation of BarA does not involve FtsH‐dependent proteolysis (Contreras et al. 2023), suggesting that the capacity of SPFH domain‐containing proteins to mediate protein–protein interactions and assemble into higher‐order complexes may enable bacteria to fine‐tune diverse physiological processes. Recent structural studies have shown that SPFH proteins can self‐assemble into higher‐order oligomers, such as rings or cage‐like architectures, suggesting a conserved structural function in organising membrane processes (Ma et al. 2022; Qiao et al. 2022; Tan et al. 2024). Despite these advances, the physiological roles of SPFH‐domain proteins in Gram‐negative species, particularly in the context of environmental adaptation or host interactions, remain poorly defined.

*Pseudomonas aeruginosa* is a metabolically versatile environmental bacterium and a major opportunistic pathogen capable of colonising a wide range of ecological niches and hosts (Moradali et al. 2017). It is a leading cause of healthcare‐associated infections, attributed to its intrinsic antibiotic resistance, adaptive stress responses and robust biofilm formation (Qin et al. 2022). Its virulence is mediated by complex regulatory networks and an arsenal of secreted factors, including quorum sensing (QS) signals, two component transduction systems, proteolytic enzymes and toxins such as pyocyanin that facilitate colonisation and immune evasion (Diggle and Whiteley 2020; Qin et al. 2022). While membrane‐associated proteins are central to the regulation of these processes, the potential roles of SPFH‐domain proteins in *P. aeruginosa* physiology and pathogenesis have not been systematically explored.

Here, we present a comprehensive analysis of the SPFH protein repertoire in *P. aeruginosa* PA14. We identified nine genes encoding SPFH‐domain‐containing proteins and examined their phylogenetic relationships, genomic organization and evolutionary conservation. To investigate their potential physiological roles, we generated single and combinatorial deletion mutants and evaluated their phenotypes in assays relevant to environmental adaptation and virulence, including biofilm formation, motility, resistance to oxidative stress and pathogenicity in the *Galleria mellonella* infection model. The selection of phenotypes analysed in this study was guided by previously reported functions of SPFH‐domain proteins in other bacteria, including their roles in signal transduction, protease modulation, secretion systems, oxidative stress resistance and virulence‐associated traits (Kihara et al. 1996, 1997; Gao et al. 2006; Schneider, Mielich‐Süss, et al. 2015; Mielich‐Süss et al. 2017; Contreras et al. 2023; Ukleja et al. 2024). Our results revealed that, despite the structural and phylogenetic diversity of the SPFH proteins, none of the tested mutants exhibited major defects in these traits. These findings indicate that SPFH proteins are largely dispensable for cell viability and virulence under the tested conditions. This work highlights the potential for functional specialisation or context‐dependent activity of SPFH‐domain proteins in Gram‐negative bacteria and lays the groundwork for future studies to elucidate their roles in membrane organization, stress adaptation and host interaction under more physiologically relevant or environmentally complex conditions.

## 2 Materials and Methods

### 2.1 Sequence Retrieval and Phylogenetic Analyses

Sequences of SPFH‐containing proteins used in this study (Table S1) were retrieved from the EcoCyc *E. coli* database (Karp et al. 2023), the Pseudomonas Genome Database (Winsor et al. 2016) and the UniProt database (Bateman et al. 2025). Proteins belonging to the Band 7/SPFH domain superfamily (IPR036013) were identified based on InterPro annotations (Blum et al. 2025). *P. aeruginosa* SPFH protein‐coding sequences, as well as their genomic organization and context, were obtained from the Pseudomonas Genome Database (Winsor et al. 2016). Multiple sequence alignments were generated using MUSCLE (Edgar 2004), and phylogenetic relationships were inferred with the Neighbour‐Joining method (Saitou and Nei 1987) implemented in MEGA version 11 (Tamura et al. 2021). The bootstrap consensus tree was constructed from 1000 replicates, and branches with less than 25% bootstrap support were collapsed.

### 2.2 Bacterial Strains, Plasmids and Growth Conditions

*P. aeruginosa* PA14 (UCBPP‐PA14) was used as the wild‐type (WT) strain and all mutant derivatives were generated from it. *E. coli* Top10 (Invitrogen) was used for routine transformation, cloning and maintenance of plasmids. *E. coli* strain S17‐1 λpir (Simon et al. 1983) was used to mobilise plasmids into *P. aeruginosa* by bacterial conjugation. *P. aeruginosa* and *E. coli* were routinely cultured at 37°C in LB medium. Solid LB medium was obtained by supplementing the broth with agar at a final concentration of 1.5% (w/v). When necessary, the growth medium was supplemented with gentamicin (20 μg/mL for *E. coli* and 100 μg/mL for *P. aeruginosa* ). Vogel–Bonner minimal medium (VBMM) agar and Pseudomonas Isolation agar (PIA) (Difco) were used for the selective growth of *P. aeruginosa* and to select against *E. coli* following biparental mating assays. No‐salt LB (NSLB) agar supplemented with 10% (w/v) sucrose was employed for the counter‐selection of *P. aeruginosa* merodiploids, enabling the isolation of specific deletion mutants after the second homologous recombination event. Pseudomonas broth (PB) (Essar et al. 1990) was used to measure pyocyanin production in liquid culture. M63 minimal medium (Pardee 1959), supplemented as indicated, was used as a salt‐based medium to grow *P. aeruginosa* for the assessment of biofilm formation and swimming motility. M9 minimal medium (Sambrook and Russell 2001), with appropriate additives, was used to evaluate swarming motility of *P. aeruginosa* strains.

### 2.3 Construction of Unmarked Deletion Mutants of *P. aeruginosa*

Unmarked deletions at 7 loci in the *P. aeruginosa* PA14 genome, corresponding to regions encoding the SPFH proteins PaFlo3, PaFlo4, PaFlo8, PaFlo9, PaFlo1–PaFlo2, PaFlo5–PaFlo6–PaFlo7 and GacA, were generated by a two‐step allelic exchange protocol using the suicide vector pEX18Gm (Hoang et al. 1998), as previously described (Hmelo et al. 2015). Briefly, DNA fragments of 500–1000 bp immediately upstream and downstream of each target locus were PCR‐amplified from PA14 genomic DNA (primers used are listed in Table S2) and assembled into pEX18Gm using the NEBuilder HiFi DNA Assembly Master Mix (NEB), following the manufacturer's instructions. The resulting suicide plasmids were verified by DNA sequencing and introduced into *P. aeruginosa* PA14 via biparental mating. For conjugation, the plasmid was first transformed into *E. coli* S17‐1, which was grown in LB medium containing 20 μg/mL gentamicin to an OD600 of 0.5–0.6. In parallel, an overnight culture of the recipient *P. aeruginosa* strain was diluted 1:2 in fresh LB and incubated at 42°C for 3–4 h. Donor and recipient cultures (1.5 and 0.5 mL, respectively) were mixed, centrifuged at 13,000*g* for 2 min and the pellet resuspended in 50 μL of LB. The mixture was spotted onto the centre of a pre‐warmed LB agar plate and incubated overnight at 30°C. Cells were then scraped from the plate, resuspended in 1 mL of phosphate‐buffered saline (PBS), pH 7.6 and 10, 100 and 200 μL aliquots were plated on VBMM or PIA agar containing 100 μg/mL gentamicin, followed by incubation for 24–72 h at 37°C. Single merodiploid colonies were streaked onto NSLB agar supplemented with 10% (w/v) sucrose and incubated for 24–48 h at 30°C. Colonies were subsequently screened by PCR to confirm the desired deletions.

### 2.4 Pyocyanin Quantification

Pyocyanin production was quantified as previously described (Essar et al. 1990), with minor modifications. Briefly, overnight cultures of each *P. aeruginosa* strain were used to inoculate 5 mL of PB medium at an initial OD600 of 0.05 and cultures were incubated for 24 h at 37°C with shaking. After recording the OD600, 1.5 mL of each culture was transferred to a microcentrifuge tube and centrifuged at 13,000*g* for 5 min. One millilitre of the supernatant was transferred to a clear tube and vigorously extracted with 0.6 mL of chloroform. Following phase separation by centrifugation, 0.5 mL of the lower chloroform phase was transferred to a fresh clear tube and extracted with 0.8 mL of 0.2 N HCl. After centrifugation, 0.65 mL of the upper aqueous phase was collected, and absorbance was measured at 520 nm using 0.2 N HCl as the blank. Pyocyanin concentrations, expressed as micrograms per millilitre of culture supernatant, were calculated by multiplying the OD520 value by 17.072 (Kurachi 1958).

### 2.5 Determination of Alkaline Protease Activity

Alkaline protease activity was quantified spectrophotometrically as previously described (Howe and Iglewski 1984). Briefly, WT and mutant *P. aeruginosa* strains were grown overnight in LB at 37°C with shaking, the OD600 was recorded and culture supernatants were collected by centrifugation. Reactions containing 0.0055–0.0065 g of Hide‐Remazol Brilliant Blue R substrate (Sigma‐Aldrich), 50 μL of supernatant and 950 μL of protease buffer (20 mM Tris–HCl, 1 mM CaCl_2_, pH 8.0) were incubated for 20 min at 37°C with shaking. The reactions were stopped on ice, samples were centrifuged at 4°C and the absorbance of the supernatants was measured at 595 nm and normalised to the OD600 of the corresponding starting culture.

### 2.6 Determination Elastase Activity

Elastase (LasB) activity was determined spectrophotometrically as described previously (Ohman et al. 1980). WT and mutant *P. aeruginosa* strains were grown overnight in LB at 37°C with shaking and the OD_600_ was recorded. Culture supernatants were obtained by centrifugation and diluted 1:10 in elastase buffer (100 mM Tris–HCl, 1 mM CaCl_2_, pH 7.0). Then, 50 μL of this dilution were added to 950 μL of elastase buffer and mixed with 0.006 g of Elastin Congo Red substrate (Sigma‐Aldrich) in a final volume of 1 mL. After incubation at 37°C with shaking for 2 h, reactions were chilled on ice and centrifuged at 4°C. The absorbance of the supernatant was measured at 495 nm and normalised to the OD_600_ of the starting culture.

### 2.7 *G. mellonella* Killing Assays

*P. aeruginosa* strains were grown overnight in LB medium at 37°C, diluted in fresh LB to an OD_600_ of 1.0, harvested by centrifugation (13,000*g*, 2 min) and resuspended in 1 mL of 0.85% (w/v) NaCl. Serial 10‐fold dilutions were prepared in 0.85% NaCl up to 10^7^‐fold. Aliquots (20 μL) of the 10^6^‐ and 10^7^‐fold dilutions were injected into *G. mellonella* larvae via the hindmost left proleg using a 0.3 mL insulin syringe. The colony‐forming units (CFUs) of these dilutions were determined on agar plates and were found to correspond to approximately 30–50 and 3–5 bacteria, respectively. In parallel, appropriate dilutions were plated on LB agar to determine CFU counts. For each strain and dilution, 10 larvae were inoculated per experiment. Infected larvae were incubated at 37°C, with survival recorded after 17 h and monitored for up to 48 h. Larvae were scored as dead when unresponsive to gentle shaking or tactile stimulation with a pipette tip. Survival data were analysed using Kaplan–Meier curves and statistical significance was assessed with the Mantel–Cox log‐rank test.

### 2.8 Motility Assays

Swarming assays were performed on freshly prepared M9 soft agar plates (0.5% w/v agar) supplemented with 0.2% glucose, 0.5% Casamino Acids, 1 mM MgSO_4_ and 1 mM CaCl_2_. Aliquots (1.5 μL) of overnight *P. aeruginosa* cultures grown in LB medium were spotted onto the plate surface and incubated at 37°C for 18 h. Swarming surface coverage was quantified using ImageJ software. Swimming motility assays were conducted on freshly prepared M63 soft agar plates (0.3% w/v agar) supplemented with 0.2% glucose, 0.5% Casamino Acids and 1 mM MgSO_4_. Aliquots (1.5 μL) of overnight *P. aeruginosa* cultures grown in LB medium were inoculated by piercing into the agar at the centre of the plate. Plates were incubated at 37°C for 24 h, after which the swimming halo diameter was measured.

### 2.9 Biofilm Formation Assays

Biofilm formation was assessed by quantifying cell adhesion to the wells of a microtiter plate, as previously described (Coffey and Anderson 2014). Briefly, overnight LB cultures were diluted to a final OD_600_ of 0.05 in fresh M63 medium supplemented with 0.2% glucose, 0.5% Casamino Acids and 0.4% arginine and 100 μL aliquots were dispensed into each well of a 96‐well microtiter plate. Plates were covered and incubated statically at 37°C for 24 h. Non‐adherent cells were then removed and the wells were gently rinsed with distilled water. Biofilms were stained with 125 μL of 0.1% (w/v) crystal violet for 10 min, followed by rinsing with distilled water. The dye bound to the biofilm was solubilised with 150 μL of 30% (v/v) acetic acid in water and the absorbance of the resulting solution was measured at 570 nm. Biofilm biomass was expressed as the ratio of OD_570_ to the OD_600_ of the corresponding overnight culture.

### 2.10 Tobramycin Sensitivity Assay

*P. aeruginosa* strains were grown overnight in LB medium at 37°C. Cultures were diluted 1:100 in fresh LB and incubated at 37°C with shaking until reaching an OD_600_ of 0.2. One millilitre of each culture was harvested by centrifugation, washed once and resuspended in 1 mL of 0.85% (w/v) NaCl. Aliquots of 8 μL from each cell suspension were spotted onto LB agar plates supplemented with tobramycin at final concentrations of 0.125, 0.25, 0.5, 1 or 2 μg/mL. Plates were incubated at 37°C for 18 h and subsequently assessed for bacterial growth.

### 2.11 Oxidative Stress Resistance Assays

Overnight cultures of *P. aeruginosa* were adjusted to an OD_600_ of 0.3 in 0.85% (w/v) NaCl. Serial 10‐fold dilutions were then prepared in 0.85% NaCl up to 10^6^‐fold. Aliquots of 10 μL from each dilution were spotted onto LB agar plates without additives or supplemented with 0.6 mM H_2_O_2_ or 0.15 mM tert‐butyl hydroperoxide (TBH). Plates were incubated at 37°C for 24 h and bacterial survival was assessed based on colony growth.

### 2.12 Statistics

All quantitative experiments were performed in at least triplicate and results are presented as mean ± standard deviation. Statistical significance was determined by one‐way analysis of variance (ANOVA) followed by Dunnett's multiple‐comparison post hoc test.

## 3 Results

### 3.1 Identification of Putative SPFH‐Domain Proteins in *P. aeruginosa*

**FIGURE 1:**
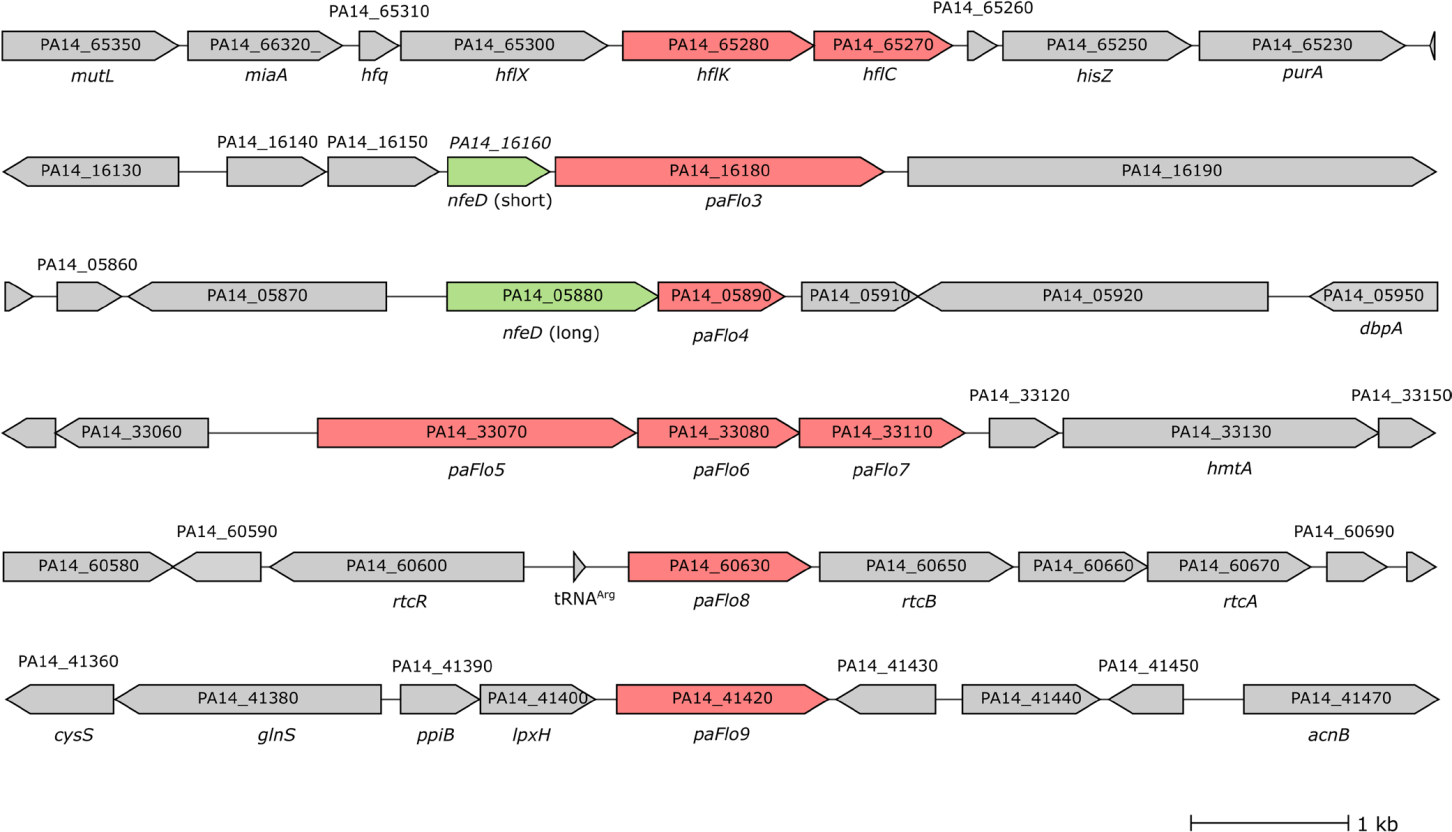
Gene organization and genomic context of putative SPFH‐domain‐containing proteins in *Pseudomonas aeruginosa* PA14. The six genomic loci of *P. aeruginosa* PA14 containing the nine putative SPFH‐domain protein‐encoding genes (designated paFlo1–paFlo9) are shown. Gene annotations are indicated within or above the corresponding arrows representing coding sequences; predicted gene names, when available, are shown below the arrows. Red arrows indicate SPFH‐domain protein‐encoding genes; green arrows indicate predicted *nfeD* genes; grey arrows represent other, unrelated genes.

The SPFH‐domain containing protein family is highly conserved across all domains of life. Bacterial genomes typically encode 1–10 SPFH‐domain containing proteins (hereafter referred to as SPFH proteins), which exhibit significant variability in their N‐ and C‐terminal regions (Hinderhofer et al. 2009). To investigate the presence of such proteins in *P. aeruginosa* , *blast* (*tblastn*) searches were conducted using the amino acid sequences of HflC, HflK, YbbK and YqiK from *E. coli* as queries against the *P. aeruginosa* PA14 genome (Winsor et al. 2016). These searches identified seven protein sequences with a putative SPFH domain. Furthermore, nine potential SPFH proteins were identified within the InterPro database (Band 7/SPFH domain superfamily, IPR036013) (Blum et al. 2025) for the *P. aeruginosa* PA14 genome, including the seven proteins previously identified through *blast* searches. A genomic locus comprising two SPFH protein‐encoding genes, *PA14_65280* and *PA14_65270*, was clearly identified as homologous to the *hflK* and *hflC* locus in *E. coli* , based on both synteny and sequence conservation of the encoded proteins. Specifically, HflK and HflC of *P. aeruginosa* share 44% identity / 60% similarity and 40% identity / 60% similarity, respectively, with HflK and HflC of *E. coli* across the full length of their amino acid sequences. The remaining seven SPFH proteins (designated PaFlo3 to PaFlo9) are dispersed across five distinct chromosomal loci in *P. aeruginosa* (Figure 1) and do not exhibit orthology to any known *E. coli* protein. Two SPFH‐encoding genes, PA14_05890 (*paFlo 4*) and PA14_16180 (*paFlo3*), form operons with genes encoding NfeD homologues PA14_16160 (short NfeD)/paFlo3 and PA14_05880 (long NfeD)/paFlo4 (Figure 1). On the other hand, we found that three SPFH proteins (PaFlo5, PaFlo6 and PaFlo7) are encoded within a single predicted operon (*PA14_33070*, *PA14_33080* and *PA14_33110*), suggesting possible structural and functional interactions. In contrast, the *PA14_60630* gene, which encodes PaFlo8, appears to be embedded within the *rtc* locus, which includes genes involved in RNA repair (RtcA, RtcB and the regulator RtcR). Finally, *PA14_41420*, encoding PaFlo9, is a monocistronic gene located adjacent to the conserved *ppiB‐lpxH* operon, which codes for a peptidyl‐prolyl isomerase and a key enzyme in lipid A biosynthesis, respectively. Altogether, the distribution and variability in the genomic contexts of the SPFH‐encoding genes suggest that these proteins may participate in distinct and specialised physiological processes in *P. aeruginosa* .

### 3.2 Evolutionary Analysis of *P. aeruginosa* SPFH Proteins

**FIGURE 2:**
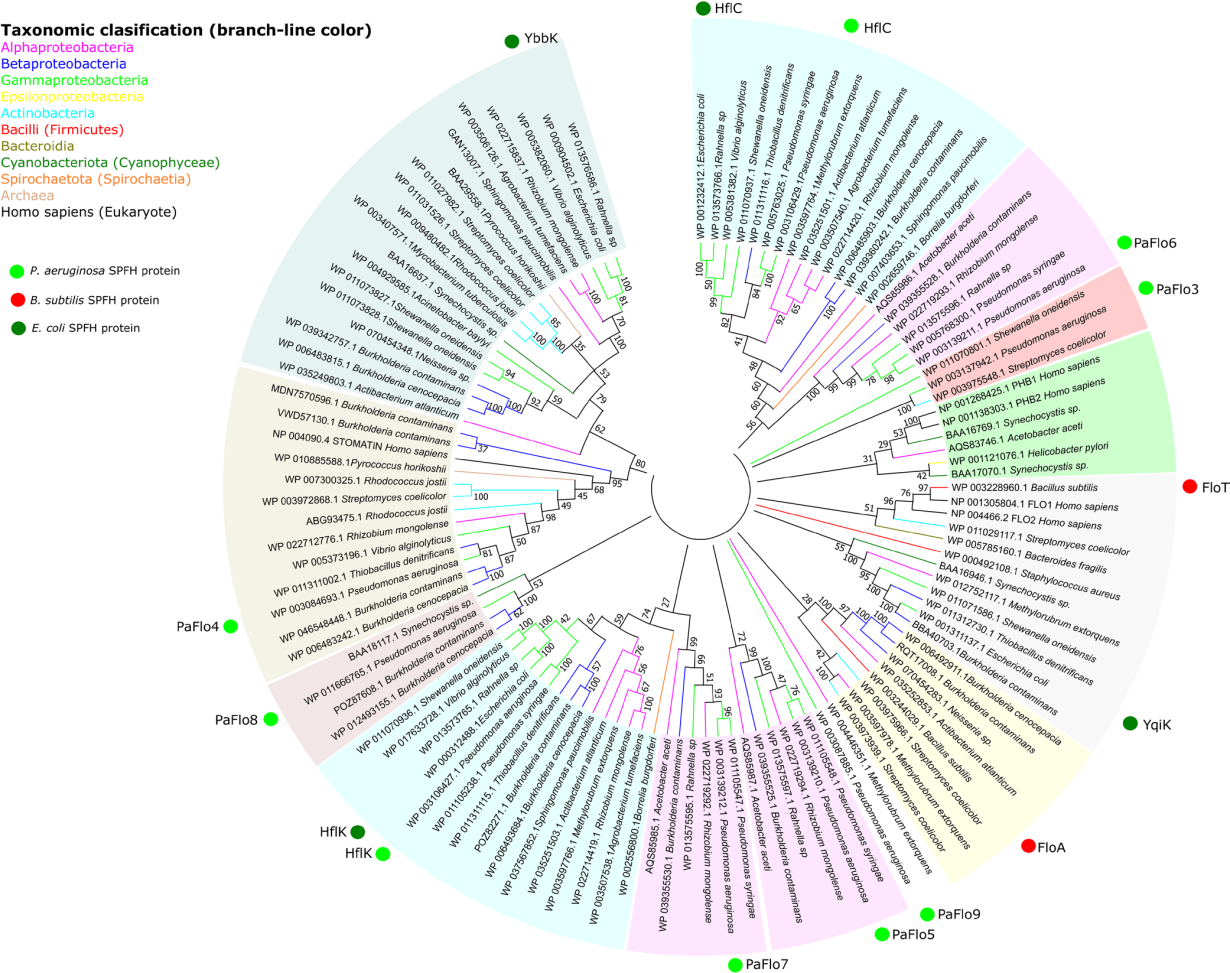
Phylogenetic analysis of selected SPFH‐domain‐containing proteins. Protein sequences were aligned using MUSCLE and an unrooted neighbour‐joining phylogenetic tree was constructed with MEGA 11. SPFH proteins were grouped according to their phylogenetic distribution, with each group shaded in a distinct colour. Groups of orthologous SPFH proteins encoded within the same operon (e.g., HflK and HflC homologues; PaFlo5, PaFlo6 and PaFlo7 homologues) share the same shading. Branches are colour‐coded according to taxonomic classification. The positions of SPFH proteins from *Pseudomonas aeruginosa*, *Escherichia coli* and *Bacillus subtilis* are highlighted with light green, dark green and blue circles, respectively.

To gain insight into the evolution and diversity of *P. aeruginosa* SPFH proteins, we performed a phylogenetic analysis including homologues from selected organisms. To this end, an unrooted, neighbour‐joining tree was inferred from nonredundant sequences, which included members of different bacterial orders and several SPFH‐domain containing eukaryotic proteins. Consistent with previous studies (Rivera‐Milla et al. 2006; Boehm et al. 2009; Hinderhofer et al. 2009), significant variations in sequence length and residue conservation among SPFH proteins posed challenges to phylogenetic analysis and hindered clear differentiation between subfamilies. Nevertheless, our analysis revealed that SPFH proteins cluster into at least 13 distinct subgroups (Figure 2). Remarkably, *P. aeruginosa* SPFH proteins are distributed across nine of these subgroups, ruling out the possibility of their origin through gene duplication events. Our analysis reveals that the operon encoding HflK and HflC homologues (highlighted in light blue in the phylogenetic tree, Figure 2) is highly conserved across multiple bacterial phyla, including γ‐proteobacteria (except Moraxellales, e.g., *Acinetobacter baylyi* ), β‐proteobacteria (except Neisseriales), α‐proteobacteria (except Rhodospirillales, such as *Acetobacter aceti* ) and Spirochaetes. Notably, in γ‐ and β‐proteobacteria, the operon includes the *hfq* and *hflX* genes (*hfq/hflX/hflK/hflC operon*), whereas in α‐proteobacteria and Spirochaetes, the operon consists solely of *hflK* and *hflC* genes (*hflK/hflC* operon). In *E. coli* , the HflK‐HflC complex has been shown to negatively regulate the ATP‐dependent zinc metalloprotease FtsH, a key component of membrane protein quality control involved in the turnover of unstable membrane proteins (Kihara et al. 1996, 1997). FtsH proteases are present in nearly all cellular organisms, with the exception of certain archaebacteria (Summer et al. 2006; Langklotz et al. 2012; Wagner et al. 2012). These proteases target a wide range of membrane‐bound and soluble proteins and participate in the control of multiple cellular processes (Ogura et al. 1999; Ito and Akiyama 2005; Janska et al. 2013; Kato and Sakamoto 2018). It is plausible that the *hflK/hflC* operon was acquired as a unit by γ‐, β‐ and α‐proteobacteria, as well as Spirochaetes, from a common ancestor, and subsequently evolved to modulate the ancestral FtsH protease. On the other hand, the phylogenetic analysis revealed that the SPFH proteins PaFlo5, PaFlo6 and PaFlo7 (highlighted in pink in Figure 2), encoded within an operon (Figure 1), are conserved among species of the order *Pseudomonadales* but are uncommon in other γ‐proteobacteria. Notably, these proteins, together with the genomic organization and context of their encoding genes, are restricted to a small subset of bacterial species from different classes. This distribution pattern suggests that the complete operon in *P. aeruginosa* and other bacteria may have originated through horizontal gene transfer. Additionally, PaFlo3, PaFlo4, PaFlo8 and PaFlo9 cluster with SPFH homologues from unrelated bacterial species but are absent in closely related species, such as those from the order *Enterobacteriales* and in the closest relative, *P. syringae* . To further characterise the distribution and genomic organization of SPFH protein‐encoding genes within the *P. aeruginosa* population, we examined the presence of the nine identified SPFH loci across 1117 fully sequenced *P. aeruginosa* genomes (Table S3). The dataset included representatives of the three major phylogenetic groups: Group 1 (including the reference strain PAO1 and the clone C strain SG17M), Group 2 (including PA14) and the more divergent Group 3, currently classified as *Pseudomonas paraeruginosa* and represented by strains such as PA7 and CR1. The collection comprised both clinical lineages and environmental isolates, enabling a comprehensive assessment of SPFH gene conservation across diverse ecological and evolutionary backgrounds. Our analysis revealed that the nine SPFH‐encoding genes are highly conserved and constitute part of the core genome of both *P. aeruginosa* and *P. paraeruginosa* (Figure S1), being detected in 99.5%–100% of the analysed genomes. Furthermore, examination of their genomic context demonstrates that the synteny of these loci is also broadly preserved across strains, indicating a remarkable degree of structural conservation in addition to sequence‐level homology. In contrast, comparative analysis across 10 reference genomes representing other *Pseudomonas* species ( *P. composti* , *P. fluorescens* , *P. knackmussii* , *P. nitroreducens* , *P. pseudoalcaligenes* , *P. putida* , *P. resinovorans* , *P. stutzeri* , *P. syringae* and *P. thermotolerans* ) revealed that only the *hflK hflC* operon is consistently conserved (Figure S2). The remaining SPFH loci displayed a markedly restricted and patchy distribution: *paFlo3* was detected exclusively in *P. pseudoalcaligenes* and *P. composti* ; the *paFlo5–paFlo6–paFlo7* operon was conserved solely in *P. knackmussii* and *P. syringae* ; *paFlo8* only in *P. nitroreducens* ; and *paFlo9* was present in *P. nitroreducens* and *P. resinovorans* . Interestingly, the gene encoding PaFlo4 appears to be present in eight of the examined *Pseudomonas* genomes, being absent only in *P. syringae* and *P. nitroreducens* , although, conservation of its surrounding genomic context was only partially observed in *P. knackmussii* . Consequently, our phylogenetic reconstruction, together with the observed patterns of distribution and synteny across *Pseudomonas* species, strongly indicate that most SPFH proteins in *P. aeruginosa* , particularly, PaFlo3–PaFlo9, were acquired through relatively recent and most likely independent horizontal gene transfer events.

### 3.3 The SPFH Proteins Are Not Essential in *P. aeruginosa* PA14

**FIGURE 3:**
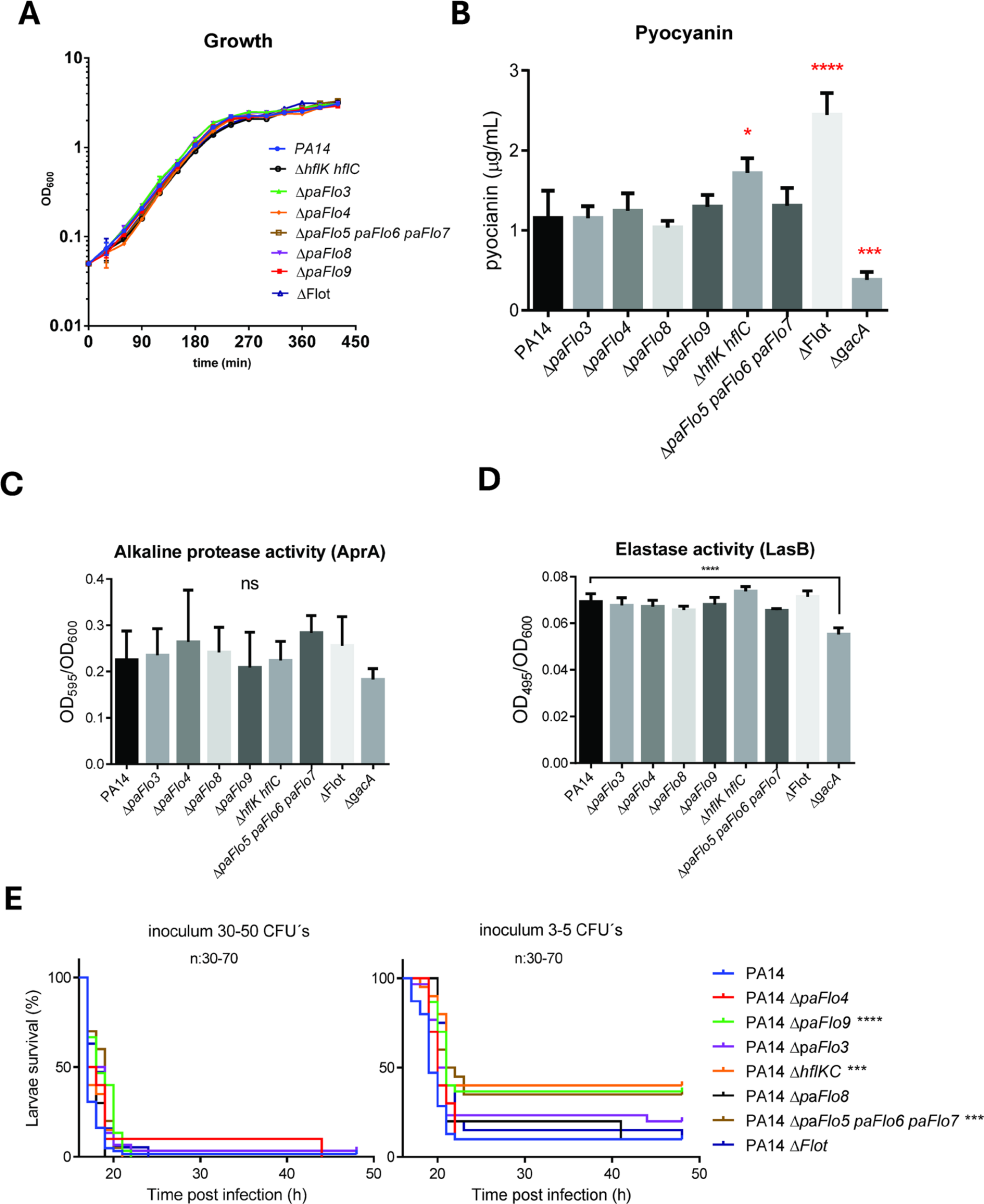
SPFH proteins of *Pseudomonas aeruginosa* are dispensable for growth, virulence factor secretion and virulence in *Galleria mellonella*. (A) Growth curves of *P. aeruginosa* PA14 (WT) and SPFH deletion mutants. (B) Pyocyanin production (μg/mL of supernatant) by PA14 (WT), Δ*hflK‐hflC*, Δ*paFlo3*, Δ*paFlo4*, Δ*paFlo5‐paFlo6‐paFlo7*, Δ*paFlo8*, Δ*paFlo9*, Δ*flot* (all SPFH‐encoding genes deleted) and Δ*gacA* strains. Quantification of extracellular (C) alkaline protease (AprA) activity and (D) elastase activity in supernatants of overnight cultures from PA14 (WT), single and multiple SPFH deletion mutants and Δ*gacA* strains. (B–D) ns, *p* > 0.05, *p < 0.05, ***p < 0.001 and ****p < 0.0001. (E) Survival of *G. mellonella* larvae infected with 30–50 CFUs (left) or 3–5 CFUs (right) of PA14 (WT) or SPFH mutants. The data were analysed using Kaplan–Meier curves and statistical significance was determined using the Mantel–Cox log‐rank test (***p < 0.001; ****p < 0.0001). *n*: number of larvae infected per strain.

To investigate the physiological roles of SPFH proteins in *P. aeruginosa* , six mutant strains were generated in the PA14 background. These mutants carried unmarked in‐frame deletions in the coding sequences of individual SPFH proteins (PaFlo3, PaFlo4, PaFlo8 or PaFlo9) or in the polycistronic regions encoding HflK/HflC or PaFlo5/PaFlo6/PaFlo7. Additionally, to explore potential functional redundancy among the nine SPFH proteins, a *P. aeruginosa* strain lacking all nine coding sequences (designated ΔFlot) was generated. The genotypes of all deletion mutants were confirmed by PCR. To determine the impact of SPFH protein absence on bacterial growth, cultures of the PA14 WT strain and its SPFH‐mutant derivatives were inoculated into LB medium at an initial optical density at 600 nm (OD_600_) of 0.05 and grown aerobically. No differences in growth rate were observed between the SPFH mutants and the WT strain (Figure 3A). These findings indicate that SPFH proteins are not essential for cell viability or aerobic growth in rich media.

### 3.4 Deletion of SPFH Proteins Does Not Significantly Impair Virulence Factor Production or *G. mellonella* Virulence

As an initial step in the functional characterisation of SPFH proteins, we investigated their impact on the production of three well‐established secreted virulence factors: pyocyanin, LasB elastase and alkaline protease (AprA). Pyocyanin, a blue redox‐active compound, is produced almost exclusively by *P. aeruginosa* (Mavrodi et al. 2001). This compound contributes to bacterial niche competition and host colonisation; consequently, pyocyanin‐deficient strains exhibit attenuated virulence (Lau et al. 2004). Pyocyanin production, which begins with the synthesis of the phenazine‐1‐carboxylic acid (PCA), is intricately regulated by multiple pathways, including the Las, Rhl and Pqs QS systems, the regulators RsaL, MvaU and RpoS and the post‐transcriptional Rsm system, which is modulated by the GacS/GacA two‐component system (Suh et al. 1999; Lapouge et al. 2008; Higgins et al. 2018; García‐Reyes et al. 2020; Fang et al. 2021). Alkaline protease (AprA) and elastase B (LasB) are critical virulence factors that degrade host immune components (Matsumoto et al. 1998) and elastin, a key component of pulmonary tissue and blood vessels (Kessler et al. 1997), respectively. The production of both proteases is governed by the QS regulatory network (Rust et al. 1996; Bleves et al. 2010). To assess the influence of SPFH protein deletion on these regulatory pathways, we measured pyocyanin production and alkaline protease and elastase activities in the *P. aeruginosa* PA14 WT strain, SPFH mutant derivatives and in a *gacA* mutant as a control. As expected, pyocyanin production was significantly reduced in the *gacA* mutant compared to the WT (Figure 3B) (Reimmann et al. 1997). Deletion of the *hflK‐hflC* operon resulted in a slight but statistically significant increase in pyocyanin secretion compared to the WT strain (Figure 3B). This observation is consistent with previous findings showing that a *P. aeruginosa ftsH* mutant produces approximately twice as much pyocyanin as its isogenic WT counterpart, SG17M (Kamal et al. 2019). Since the FtsH activity is regulated by the HflK‐HflC complex and it has been reported that FtsH target PhzC, an enzyme required for the production of phenazine‐1‐carboxylic acid (PCA), the immediate precursor of pyocyanin, it is plausible that the increased pyocyanin levels observed in the *hflK‐hflC* mutant are mediated by altered FtsH activity (Kamal et al. 2019). Notably, the complete deletion of all nine genes encoding SPFH proteins led to a two‐fold increase in pyocyanin production relative to the WT. However, no significant differences in pyocyanin production were observed in the *paFlo3*, *paFlo4*, *paFlo8*, *paFlo9* and *paFlo5‐paFlo6‐paFlo7* mutants compared to the WT (Figure 3B). Furthermore, alkaline protease and elastase activities were comparable between the supernatants of WT and all SPFH single and multiple mutant cultures (Figure 3C,D), suggesting that SPFH proteins do not significantly affect the production, regulation or secretion of AprA or LasB.

To evaluate the impact of SPFH protein deletion on virulence, we employed the *G. mellonella* larval infection model, a widely established non‐mammalian host system for studying microbial pathogenesis (Jander et al. 2000; Miyata et al. 2003; Wojda et al. 2020). This model is particularly suited for assessing Type III secretion system (T3SS)‐dependent virulence in *P. aeruginosa* , because impairment of T3SS function significantly reduces larval mortality (Miyata et al. 2003). Larvae were infected with 30–50 CFUs of PA14 or SPFH‐deletion mutants. Under these conditions, infection with PA14 or any mutant strain caused 100% mortality within 21 h (Figure 3E), confirming the high pathogenicity of *P. aeruginosa* to *G. mellonella* (Jander et al. 2000; Axline et al. 2025) and indicating that SPFH proteins are not essential for virulence under high‐inoculum conditions. To assess whether more subtle differences in virulence could be detected, the inoculum was reduced to approximately three to five CFUs per larva. Under these conditions, the *paFlo1‐paFlo2*, *paFlo9* and *paFlo4‐5‐6* mutants showed a modest but reproducible decrease in lethality, with 30%–40% of larvae surviving at 48 h (Figure 3E). In contrast, the Δ*Flot* mutant displayed virulence comparable to the WT strain, suggesting potential compensatory effects among SPFH proteins. It has to be noted that reproducible delivery of very low bacterial doses is technically challenging and may introduce variability in survival curves. Overall, our findings indicate that SPFH proteins in *P. aeruginosa* do not appear to play a direct or substantial role in virulence‐related processes.

### 3.5 Effect of the Absence of SPFH‐Containing Proteins on Motility and Biofilm Formation

*P. aeruginosa* can move in liquid environments, on semi‐solid media and across solid surfaces via swimming, swarming and twitching motility, respectively. Swimming, which occurs in aqueous environments and low agar concentrations, relies solely on a functional polar flagellum, while twitching motility is mediated by Type IV pili (Rashid and Kornberg 2000; Whitchurch et al. 2004; Burrows 2012). Swarming, on the other hand, a complex and social form of motility, involves the rapid and coordinated movement of bacterial cells and requires flagella, Type IV pili and the production of biosurfactants such as rhamnolipids and 3‐(3‐hydroxyalkanoyloxy)alkanoic acids (HAAs) (Overhage et al. 2007; Tremblay et al. 2007).

**FIGURE 4:**
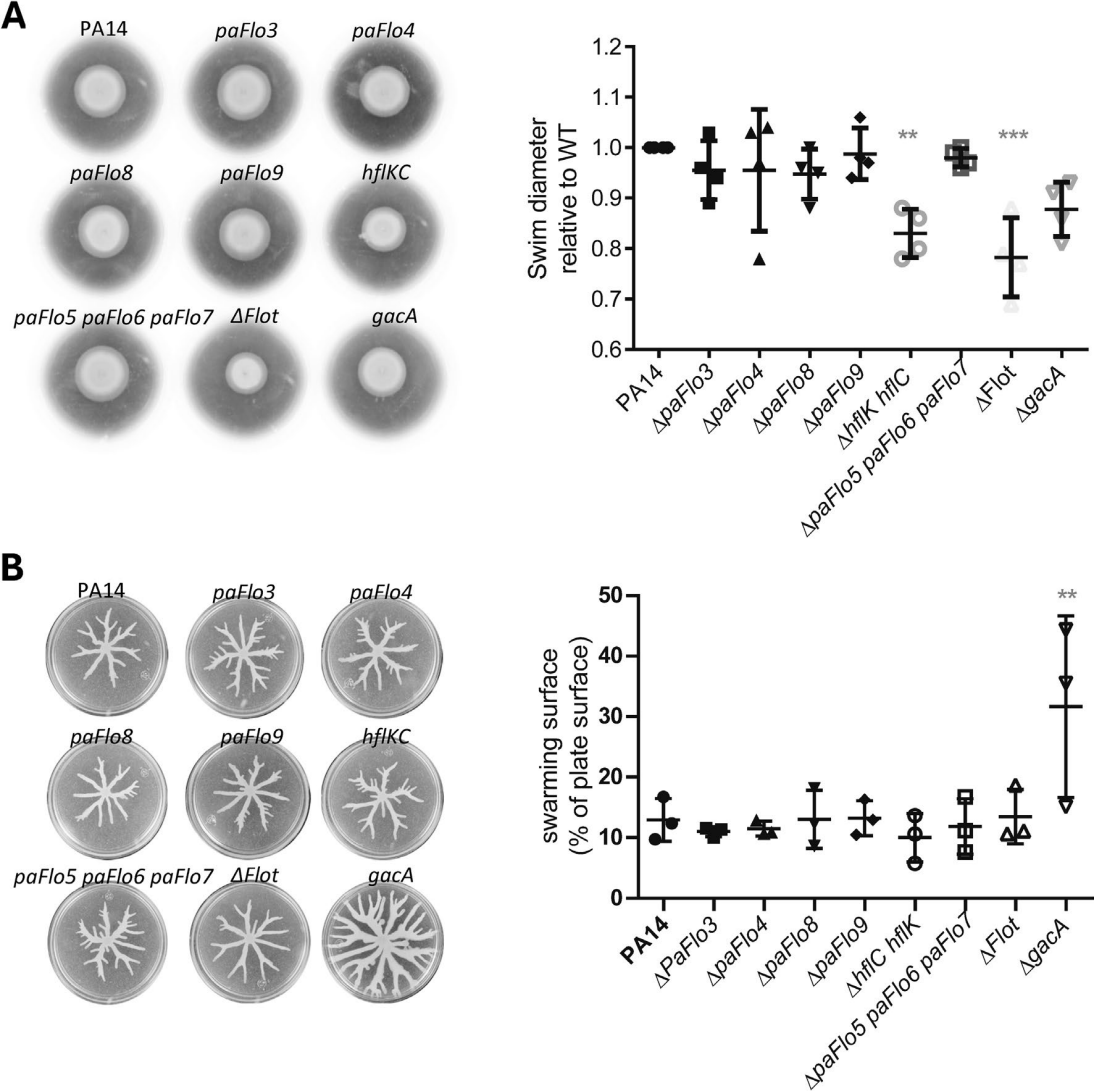
Deletion of genes encoding SPFH‐domain‐containing proteins does not affect swimming nor swarming motility in *Pseudomonas aeruginosa*. (A) Left panel, representative images of the swimming of the PA14 strain and its mutant derivatives. Right panel, quantification of swim diameters of SPFH and *gacA* mutants, relative to the wild type. Bars show means ± SD, with individual measurements shown; **p < 0.01, ***p < 0.001. (B) Left panel, representative images of the swarming of the PA14 strain and its mutant derivatives. Right panel, percentage of swarm surface occupancy of SPFH and *gacA* mutants, relative to that of the wild type. Bars show means ± SD, with individual measurements shown; **p < 0.01.

To investigate potential alterations in flagellar function, Type IV pili activity and/or biosurfactant production resulting from single or multiple SPFH‐encoding gene deletions, we assessed the swimming and swarming motility of mutant strains compared to the PA14 WT strain. For swimming assays, *P. aeruginosa* PA14 and its derivative mutant strains were point‐inoculated at the centre of M63 soft agar plates (0.3% agar) and incubated at 37°C for 16 h. All PA14‐derived strains exhibited outward swimming with concentric chemotactic ring formation, indicating that deletion of SPFH proteins did not abolish flagellar function. The *gacA* mutant strain, used as a control, showed swimming behaviour comparable to that of the WT strain, consistent with previous reports (Heurlier et al. 2004; Hassan et al. 2010). Similarly, no significant differences in swimming diameters were observed for the *paFlo3*, *paFlo4*, *paFlo8*, *paFlo9* and *paFlo5‐paFlo6‐paFlo7* deletion mutants compared to the PA14 strain. In contrast, a slight but statistically significant reduction in swimming diameter was observed for the *hflK hflC* (17% reduction) and ∆*Flot* (22% reduction) mutants relative to WT (Figure 4A). This reduced swarming motility in the *hflK‐hflC* mutant may result from altered FtsH activity, given that a comparable swimming defect was previously described in an *ftsH* mutant (Kamal et al. 2019). For swarming assays, overnight cultures of PA14, *gacA* and SPFH mutant strains were inoculated onto M8‐CAA‐glucose plates containing 0.5% agar and incubated at 37°C for 16 h. Under these conditions, PA14 cells exhibited characteristic dendritic expansion across the surface, covering approximately 12% of the plate. As reported, the *gacA* mutant displayed enhanced swarming motility, covering around 30% of the plate surface, consistent with its known hyper‐swarming phenotype (Yeung et al. 2009; Fadel et al. 2022). However, none of the individual SPFH deletion mutants, nor the strain lacking all nine SPFH proteins, showed any notable alteration in swarming motility (Figure 4B). These results suggest that SPFH proteins do not play a major role in the regulation or function of flagella, Type IV pili or biosurfactant production in *P. aeruginosa* .

**FIGURE 5:**
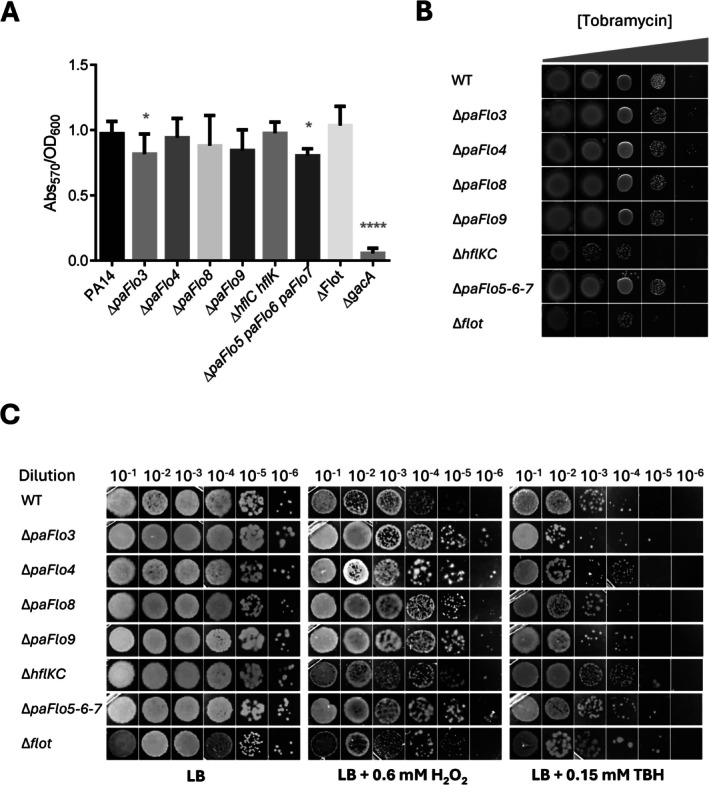
Biofilm formation, oxidative stress survival and intrinsic tobramycin susceptibility of SPFH deletion mutants. (A) Quantification of biofilm formation by PA14 (WT) and SPFH deletion mutants, measured in 96‐well microtiter plates under static conditions using crystal violet staining. The Δ*gacA* mutant was included as a control. *p < 0.05, ****p < 0.0001. (B) Representative images from tobramycin sensitivity assays. Cells of *Pseudomonas aeruginosa* PA14 (WT) and of SPFH deletion mutants were grown to OD600 = 0.2, washed and spotted onto LB agar plates supplemented with 0.125, 0.25, 0.5, 1 or 2 μg/mL tobramycin. (C) Representative images from survival assays of serially diluted *P. aeruginosa* PA14 and SPFH mutants. Dilutions were spotted on LB agar plates without additives (control) or supplemented with hydrogen peroxide (H_2_O_2_) or tert‐butyl hydroperoxide (TBH), at the indicated concentrations.

Then, we explored biofilm formation, another social and tightly regulated behaviour of *P. aeruginosa* . Biofilms are organised communities of bacterial cells encased in an extracellular matrix composed of polymeric substances, including polysaccharides, proteins, nucleic acids and lipids, that adhere to a solid surface. The complex regulation of biofilm formation involves QS and the GacS/GacA‐Rsm regulatory network (Wei and Ma 2013). To determine whether SPFH proteins play a role in biofilm development, we performed biofilm formation assays using PA14 and the *hflK*‐*hflC*, *paFlo3*, *paFlo4*, *paFlo5‐paFlo6‐paFlo7*, *paFlo8*, *paFlo9*, Δ*Flot* and *gacA* (as a control) mutant strains. As expected, deletion of *gacA* resulted in a significant decrease (20‐fold) in biofilm formation (Figure 5A) (Yeung et al. 2009; Chambonnier et al. 2016). Deletion of *hflK‐hflC*, *paFlo4*, *paFlo8* and *paFlo9* had no detectable effect. Finally, the *paFlo3* and *paFlo5*‐*paFlo6*‐*paFlo7* triple mutants produced marginally less biofilm than the WT (approximately 20% reduction) (Figure 5A), which, although statistically significant, suggests a limited role for these SPFH proteins in biofilm formation or its regulation. Notably, *paFlo3* corresponds to *PA14_16180*, located at the distal end of the previously described biofilm‐associated cluster (BAC). This cluster has been reported to exhibit pronounced biofilm and virulence defects; however, these studies primarily focused on upstream BAC genes (e.g., *bacA*/*bacB*) rather than *PA14_16180* (Macé et al. 2008; Wallart et al. 2024). Taken together, these findings suggest that SPFH proteins are not essential for the core machinery involved in motility or biofilm development and may instead participate in other, more specialised cellular processes.

### 3.6 Effect of SPFH Protein Deletion on Intrinsic Tobramycin Resistance

Aminoglycosides such as tobramycin inhibit bacterial protein synthesis by binding to the 30S ribosomal subunit, resulting in mistranslation and accumulation of aberrant proteins. The membrane protease FtsH, and to a lesser extent its modulators HflK and HflC, contribute to intrinsic aminoglycoside resistance in *P. aeruginosa* (Hinz et al. 2011). We, therefore, evaluated tobramycin susceptibility in the SPFH mutants to explore potential links between SPFH‐domain proteins and FtsH‐dependent processes. As expected, the *hflK‐hflC* mutant exhibited a slight increase in tobramycin sensitivity compared to the WT strain (Figure 5B). A similar increase in susceptibility was observed in the Δ*flot* strain lacking all SPFH‐domain proteins. In contrast, none of the individual *paFlo* mutants displayed detectable differences in growth relative to WT across the tested tobramycin concentrations. Together, these results confirm that loss of HflKC modestly increases susceptibility to tobramycin, consistent with its role as a modulator of FtsH. They also suggest that the remaining SPFH proteins do not contribute to intrinsic aminoglycoside resistance under the conditions tested.

### 3.7 SPFH Proteins Are Not Essential for Bacterial Survival Under Oxidative Stress Conditions

A recent study reported that FloA, the sole SPFH‐domain‐containing protein in the pathogen *S. aureus* (MRSA), contributes to resistance against oxidative stress (Ukleja et al. 2024). Similarly, earlier work showed that a *so1377* knockout mutant of *Shewanella oneidensis* , lacking one of its six SPFH proteins, exhibited increased sensitivity to hydrogen peroxide compared to the WT strain (Gao et al. 2006). In contrast, another study found no significant differences in oxidative stress susceptibility between the WT strain and single or multiple deletion mutants of the five SPFH‐encoding genes in the cyanobacterium *Synechocystis* sp. (Boehm et al. 2009). To evaluate whether SPFH proteins contribute to oxidative stress resistance in *P. aeruginosa* , we evaluated the survival of the PA14 WT strain and various SPFH deletion mutants upon exposure to hydrogen peroxide (H_2_O_2_) and TBH, two well‐characterised oxidative stress‐inducing agents. Overnight cultures of PA14 and its isogenic *hflK‐hflC*, *paFlo3*, *paFlo4*, *paFlo5*‐*paFlo6*‐*paFlo7*, *paFlo8*, *paFlo9* and Δ*flot* mutants were diluted in 0.9% NaCl to an OD_600_ of 0.3 and subjected to tenfold serial dilutions up to 10^−6^. Subsequently, 10 μL of each dilution was spotted onto agar plates containing either 0.6 mM H_2_O_2_ or 0.15 mM TBH and incubated at 37°C for 24 h. Our results showed no significant differences in growth between any of the mutants and the PA14 WT strain under either stress condition (Figure 5C). These findings suggest that the SPFH proteins of *P. aeruginosa* are largely dispensable for bacterial survival under the oxidative stress conditions tested.

## 4 Discussion

In this study, we identified nine SPFH‐domain‐containing proteins in *P. aeruginosa* (*hflK*, *hflC* and paFlo3 to PaFlo9), revealing notable diversity in their genomic organization, sequence divergence and phylogenetic distribution. The variability in their genomic contexts and the low sequence conservation among these proteins suggest that they may have evolved independently or been acquired through horizontal gene transfer. Our functional analyses indicate that SPFH proteins are not essential for growth, swarming or swimming motility, biofilm formation, resistance to oxidative stress or virulence in the *G. mellonella* infection model under the conditions tested in this study. Instead, several SPFH mutants displayed subtle phenotypes, including increased pyocyanin production in the *hflK‐hflC* mutant; reduced biofilm formation in the *paFlo3* and *paFlo5‐paFlo6‐paFlo7* mutants; decreased swimming motility and enhanced tobramycin sensitivity in the *hflK‐hflC* mutant; and attenuated virulence in the *G. mellonella* model for the *hflK‐hflC*, *paFlo5‐paFlo6‐paFlo7* and *paFlo9* mutants.

Contrary to recent suggestions (Álvarez‐Mena et al. 2025), our phylogenetic analysis revealed that none of the nine SPFH proteins in *P. aeruginosa* cluster with FloA or FloT from *B. subtilis* , nor with FloA from *S. aureus* . This observation excludes a specific orthologous relationship between the SPFH‐domain proteins of these phylogenetically distant bacteria. Furthermore, most PaFlo proteins are not widely conserved across γ‐Proteobacteria or even among other *Pseudomonas* species, supporting a model of species‐specific gene content and functional divergence. The absence of conserved operonic organization and the heterogeneity of flanking genes further suggest that these SPFH proteins do not participate in a common, conserved cellular pathway, but rather fulfil distinct roles that may depend on specific environmental or physiological contexts.

Previous research on bacterial SPFH‐domain proteins has largely focused on Gram‐positive bacteria, particularly using *B. subtilis* as a model, which produces the flotillin‐like proteins FloA and FloT (Donovan and Bramkamp 2009; López and Kolter 2010). As a result, much less is known about the function of these proteins in Gram‐negative species, particularly regarding their potential roles in virulence, physiology or membrane microdomain organization. In *E. coli* , the best‐characterised SPFH‐domain proteins are HflK and HflC, which form the HflKC complex. This complex interacts with the essential membrane‐bound metalloprotease FtsH, modulating its proteolytic activity (Kihara et al. 1996, 1997, 1998; Saikawa et al. 2004). FtsH plays a central role in maintaining membrane protein homeostasis by degrading misfolded proteins and regulating key cellular processes through the selective degradation of specific folded substrates. Although only 21 FtsH substrates have been confirmed to date, comprising both soluble and membrane‐associated proteins (Bittner et al. 2017), its physiological importance is underscored by its essentiality in *E. coli* and other proteobacteria, as well as its evolutionary conservation across bacterial species and organelles such as mitochondria and chloroplasts (Tatsuta and Langer 2009; Janska et al. 2010, 2013; Nishimura et al. 2016). Previous studies have shown that, similar to *E. coli* , the FtsH protease in *P. aeruginosa* also interacts with and is regulated by the HflKC complex (Hinz et al. 2011; Kamal et al. 2019; Mawla et al. 2024). It is therefore plausible that the partial phenotypes observed in the *hflK hflC* mutant, such as reduced swimming motility, increased pyocyanin production, augmented tobramycin susceptibility, and decreased lethality in the *G. mellonella* infection model, may result from altered FtsH activity in the absence of its regulatory complex.

Recent structural studies have provided new insight into SPFH protein complexes. The supramolecular structure of the HflKC–FtsH complex was resolved, revealing a circular cage‐like assembly composed of 12 HflKC dimers anchored in the inner membrane. This structure encloses four FtsH hexamers, which interact with the inner surface of the cage and protrude into the periplasm (Ma et al. 2022; Qiao et al. 2022; Ghanbarpour et al. 2025). Other SPFH‐domain proteins have also been shown to form ring‐ or basket‐like oligomers of various sizes and conformations (Boehm et al. 2009; Takekawa et al. 2019; Yokoyama and Matsui 2023; Fu and MacKinnon 2024; Tan et al. 2024; Stoner et al. 2025). For example, the recently resolved structure of the *E. coli* YbbK–YbbJ complex, formed by the SPFH protein YbbK and the long NfeD protein YbbJ, reveals a cage‐like structure composed of 26 heterodimers that protrude into the cytoplasm (Tan et al. 2024). Despite these structural insights, functional studies indicate that SPFH proteins are not functionally redundant and may play diverse physiological roles. Other mutational and biochemical analyses have implicated bacterial SPFH proteins in several processes, including oxidative stress resistance (Gao et al. 2006; Ukleja et al. 2024), ion or metal metabolism (Yokoyama and Matsui 2005; Gao et al. 2006) and signal transduction (López and Kolter 2010; Schneider, Mielich‐Süss, et al. 2015). In *S. aureus* , FloA forms complexes with its operon partner NfeD and functions in ATP‐independent stabilisation of unfolded proteins during stress, a role essential for oxidative stress resistance and bacterial viability during infection (Ukleja et al. 2024). In contrast, our results showed that deletion of individual or multiple *paFlo* genes in *P. aeruginosa* did not lead to pronounced phenotypic changes in key virulence and stress‐related traits. The lack of drastic phenotypes suggests that PaFlo proteins may have conditionally active or highly specialised roles or that their functions are masked under the experimental conditions tested. This underscores the need to assess SPFH protein function under diverse and physiologically relevant conditions. In this regard, competitive approaches such as IVET or STM in complex habitats may help reveal subtle or niche‐specific contributions to bacterial fitness.

Taken together, our findings indicate that the SPFH proteins of *P. aeruginosa* constitute a phylogenetically diverse group whose molecular roles remain insufficiently defined. Exploring environmental conditions and integrating interactome analyses will be essential to uncover the biological networks in which these proteins participate and to better understand their contribution to bacterial physiology and pathogenesis.

## Author Contributions

**Víctor Manuel García‐Maldonado:** investigation, validation, writing – original draft, formal analysis, visualisation. **Claudia Rodríguez‐Rangel:** investigation, methodology. **Dimitris Georgellis:** writing – review and editing, supervision, conceptualisation, resources. **Adrián F. Alvarez:** conceptualization, funding acquisition, writing – original draft, writing – review and editing, validation, visualisation, formal analysis.

## Funding

This work was supported by the Dirección General de Asuntos del Personal Académico (DGAPA), Universidad Nacional Autónoma de México (UNAM) (Grants IN208721 and IN213724) and Secretaría de Ciencia, Humanidades, Tecnología e Innovación (SECIHTI), Mexico (Grant CBF2023‐2024‐1369).

## Conflicts of Interest

The authors declare no conflicts of interest.

## Supporting information


**Figure S1:** Conservation of the genomic context of SPFH loci in reference *Pseudomonas aeruginosa* strains.
**Figure S2:** Conservation of SPFH loci across non‐aeruginosa *Pseudomonas* species.

**Table S1:** NCBI reference sequence accessions of SPFH proteins used in phylogenetic analysis.
**Table S2:** Oligonucleotides used in this study.
**Table S3:** Complete *Pseudomonas aeruginosa* genomes used for occurrence and synteny analyses.

## Data Availability

The data that supports the findings of this study are available in the Supporting Information of this article.
