# Supplementary material for: Exploring the Diversity and Function of the SPFH‐Domain Containing Proteins in Pseudomonas aeruginosa
Source: Environ Microbiol Rep. 2026 Apr 16;18(2):e70340. doi: 10.1111/1758-2229.70340 (PMC13086221; doi:10.1111/1758-2229.70340)
Supplement: Supplementary file 1 — Figure S1: Conservation of the genomic context of SPFH loci in reference Pseudomonas aeruginosa strains. Figure S2: Conservation of SPFH loci across non‐aeruginosa Pseudomonas species. [file EMI4-18-e70340-s001.pptx]

## Slide 1
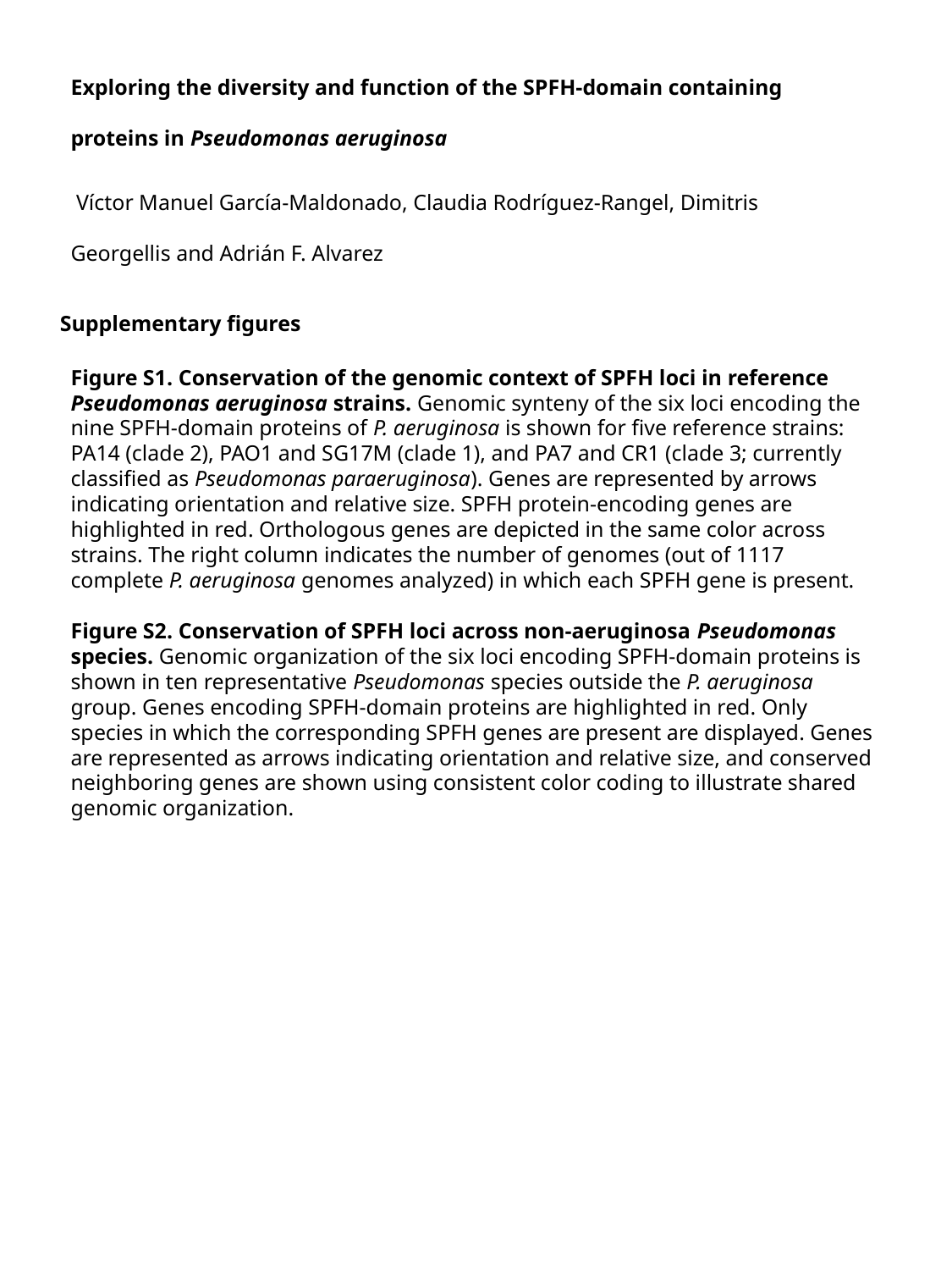

Exploring the diversity and function of the SPFH-domain containing proteins in Pseudomonas aeruginosa
 Víctor Manuel García-Maldonado, Claudia Rodríguez-Rangel, Dimitris Georgellis and Adrián F. Alvarez
Supplementary figures
Figure S1. Conservation of the genomic context of SPFH loci in reference Pseudomonas aeruginosa strains. Genomic synteny of the six loci encoding the nine SPFH-domain proteins of P. aeruginosa is shown for five reference strains: PA14 (clade 2), PAO1 and SG17M (clade 1), and PA7 and CR1 (clade 3; currently classified as Pseudomonas paraeruginosa). Genes are represented by arrows indicating orientation and relative size. SPFH protein-encoding genes are highlighted in red. Orthologous genes are depicted in the same color across strains. The right column indicates the number of genomes (out of 1117 complete P. aeruginosa genomes analyzed) in which each SPFH gene is present.
Figure S2. Conservation of SPFH loci across non-aeruginosa Pseudomonas species. Genomic organization of the six loci encoding SPFH-domain proteins is shown in ten representative Pseudomonas species outside the P. aeruginosa group. Genes encoding SPFH-domain proteins are highlighted in red. Only species in which the corresponding SPFH genes are present are displayed. Genes are represented as arrows indicating orientation and relative size, and conserved neighboring genes are shown using consistent color coding to illustrate shared genomic organization.

## Slide 2
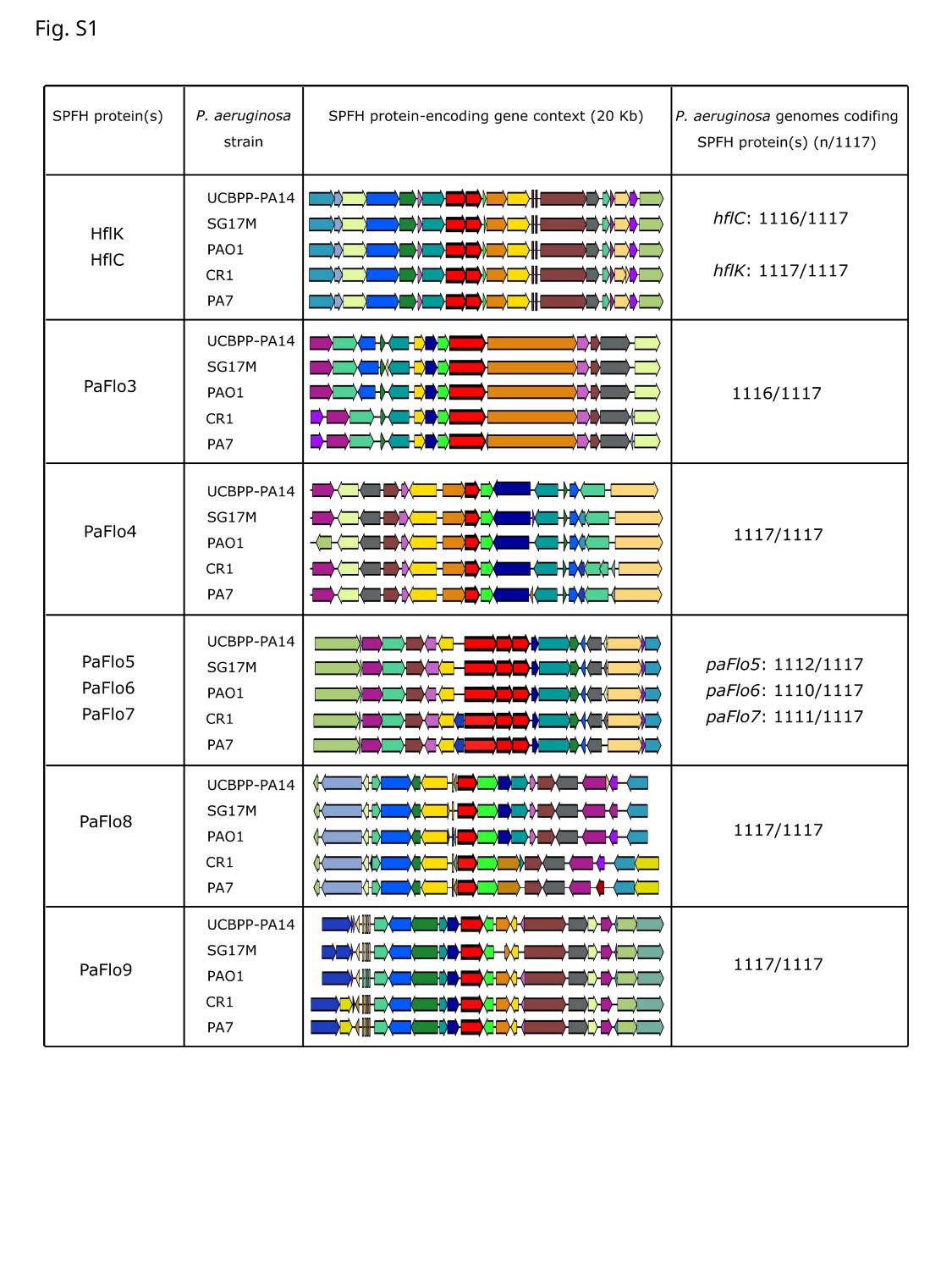

Fig. S1

## Slide 3
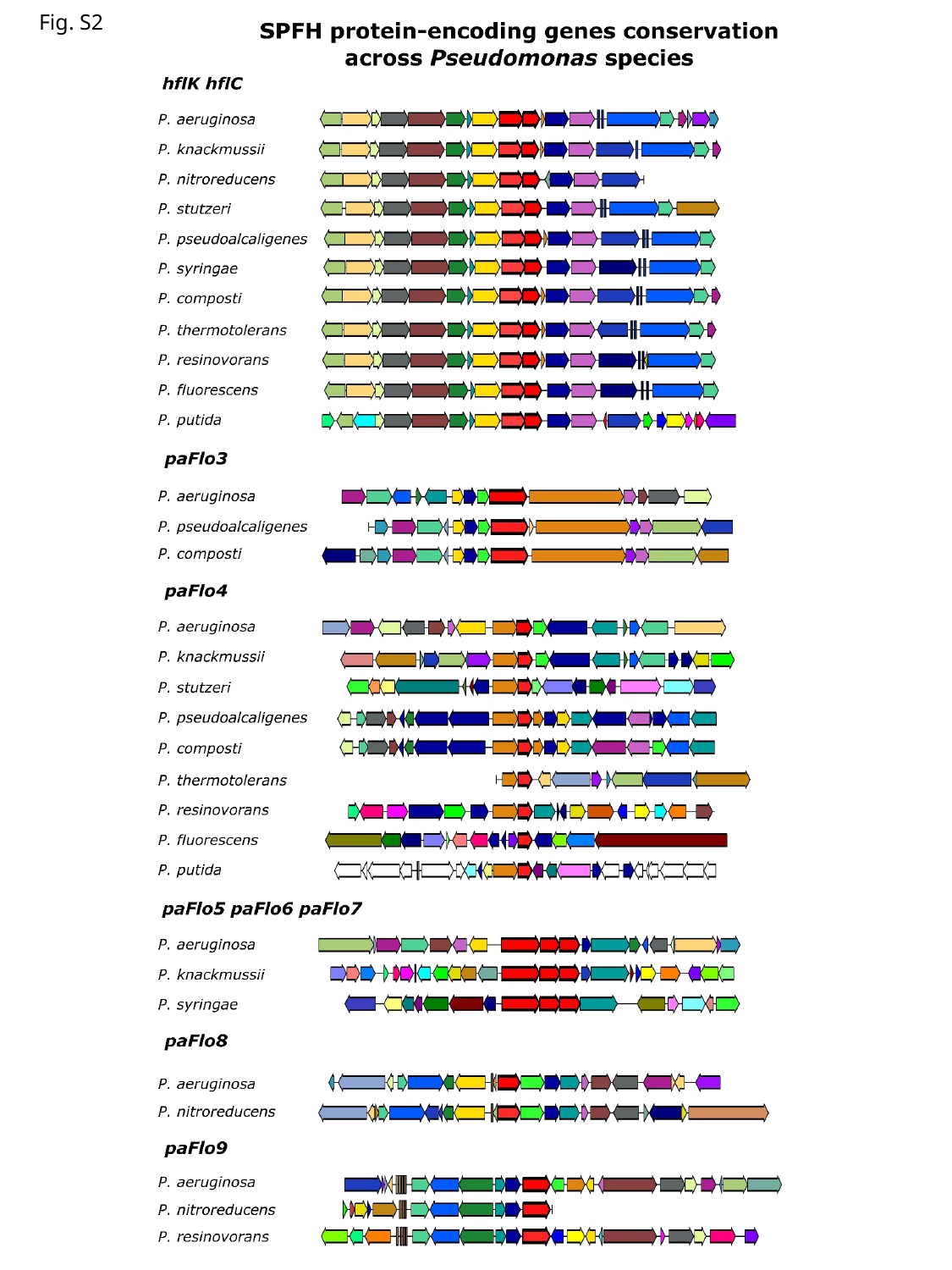

Fig. S2
